# Explainable AI in Pharmaceutics: Grad-CAM Analysis of Surface Dissolution Imaging Using Convolutional Neural Networks

**DOI:** 10.3390/pharmaceutics18040481

**Published:** 2026-04-14

**Authors:** Abdullah Al-Baghdadi, Adam Pacławski, Jakub Szlęk, Aleksander Mendyk

**Affiliations:** Department of Pharmaceutical Technology and Biopharmaceutics, Jagiellonian University Medical College, 30-688 Kraków, Poland; abdullah@alumni.uj.edu.pl (A.A.-B.); j.szlek@uj.edu.pl (J.S.); aleksander.mendyk@uj.edu.pl (A.M.)

**Keywords:** drug dissolution, convolutional neural networks, Grad-CAM, surface dissolution imaging, machine learning, solid dosage forms

## Abstract

**Background:** The dissolution of oral solid dosage forms is a key determinant of drug bioavailability, yet traditional testing methods do not capture the real-time surface dynamics of drug release. This study introduces a novel framework combining surface dissolution imaging (SDi2) with an interpretable, dual-wavelength convolutional neural network (CNN) to predict and understand dissolution behavior. **Methods:** Eight tablet formulations containing acetylsalicylic acid, sodium salicylate, or salicylamide, combined with either lactose or methylcellulose, were analyzed under two distinct, compendial conditions (pH 1.2 and pH 6.8). **Results:** Our final CNN model, which synergistically processes spectral images (280 nm for API release and 520 nm for structural changes), temporal data, and formulation composition, accurately predicted dissolution profiles, achieving a coefficient of determination of 0.89 and a root mean square error (RMSE) of 11.57. To overcome the “black-box” nature of deep learning, we employed Gradient-weighted Class Activation Mapping (Grad-CAM) to interpret the model’s predictions. The analysis revealed that the model focused on tablet edges at 280 nm, consistent with surface dissolution, and on bulk regions at 520 nm, reflecting structural changes including erosion and gel-layer growth. **Conclusions:** These findings suggest that integrating real-time imaging with explainable AI methods can support better understanding of dissolution processes in pharmaceutical formulation development.

## 1. Introduction

The dissolution of oral solid dosage forms is a critical determinant of drug bioavailability, as it governs the rate at which an active pharmaceutical ingredient becomes available for absorption. While dissolution is most evidently rate-limiting for poorly water-soluble compounds, as formalized by the Biopharmaceutics Classification System (BCS) [1], it can equally govern absorption across all BCS classes whenever intrinsic dissolution rate or excipient matrix effects limit drug presentation to the intestinal membrane [2]. Consequently, optimizing and controlling this process is a cornerstone of pharmaceutical formulation development.

Standard dissolution testing methods, governed by pharmacopeial standards, rely on bulk sampling of dissolved drug at specified intervals. While invaluable for quality control, they lack the spatial and temporal resolution needed to capture dynamic interfacial phenomena such as surface erosion, gel-layer formation, or microenvironmental pH shifts within the dissolving matrix [3]. This limitation can make it challenging to understand the behavior of dosage form in dissolution process. For example, hydrophilic polymers like methylcellulose (MC), hydroxypropyl cellulose (HPC), and hydroxypropyl methylcellulose (HPMC) are known to retard drug release by forming a viscous gel layer, a phenomenon that standard methods struggles to track [4]. Conversely, soluble fillers such as lactose promote rapid liquid penetration through the compact pore network, resulting in fast, surface-initiated dissolution [5,6].

For example, a study on HPMC matrix tablets containing diclofenac sodium and paracetamol demonstrated how swelling and gel layer formation varied with pH, directly influencing drug release rates. Diclofenac tablets formed a white, non-transparent layer at low pH due to precipitation, while a transparent gel layer developed at higher pH levels. Paracetamol tablets showed consistent swelling across all pH conditions, highlighting the complexity of dissolution behavior and the limitations of standard testing methods in capturing these dynamics [7]. Similarly, a study on 3D-printed polyethylene oxide (PEO) tablets loaded with propranolol hydrochloride revealed that drug concentration significantly affected swelling and gel layer formation. Tablets with higher drug loading exhibited increased swelling and thicker gel layers, which slowed drug release. In contrast, tablets with lower drug content showed reduced swelling and faster dissolution. Dissolution imaging provided data that conventional methods could not capture [8].

Matrix effects alone can create pronounced pH-dependent release in dissolution media. Excipient behavior like gel-layer formation, swelling, and erosion can govern dissolution kinetics independently of API ionization state, as demonstrated in polymer-matrix systems where tuning excipient composition reshapes release profiles without biorelevant surfactants or bile salts [9,10].

Recent advances in real-time imaging, particularly surface dissolution imaging (SDi2), open new opportunities for analysis of the dissolution process. The SDi2 apparatus (Pion) combines UV-Vis spectroscopy with high-resolution video capture to monitor dissolution at the tablet-medium interface, generating spatially resolved data on drug release and structural changes [11]. However, the primary challenge lies in the volume and complexity of the data generated, thousands of high-resolution frames per experiment, which are impractical to interpret manually. Traditional mathematical models, such as Korsmeyer–Peppas or Weibull equations, are insufficient in this context, as they rely on simplified assumptions about release geometry and cannot capture the spatial heterogeneity visible in imaging data [12]. Convolutional neural networks (CNNs) are well-suited for this task, offering a method to automatically extract salient features from imaging data and model dissolution kinetics [13,14]. While their application to pharmaceutical dissolution remains nascent compared to established domains such as medical imaging and materials science [15]. Proof-of-concept studies have demonstrated the feasibility of tracking tablet disintegration in real time and correlating dissolution kinetics with spectral imaging data. CNNs have also been explored for indirect prediction of dissolution behavior by analyzing structural features such as porosity maps or molecular descriptors [16]. Key challenges remain, including the scarcity of labelled datasets and the dominance of regulatory-mandated analytical methods such as UV-Vis spectroscopy and HPLC. Nevertheless, these limitations reinforce the need for interpretable, image-driven models that can complement conventional dissolution testing approaches [17,18].

A key barrier to adoption is the “black-box” nature of CNNs, which complicates regulatory acceptance and mechanistic interpretation. Gradient-weighted Class Activation Mapping (Grad-CAM) is a technique for visualizing regions in an image that influence a neural network’s predictions by using gradients from a target class to weight activation maps [19]. Unlike Class Activation Mapping (CAM), which requires a specific architecture, Grad-CAM works with any CNN. Other methods like Guided Backpropagation, Layer-wise Relevance Propagation (LRP), and Integrated Gradients offer different trade-offs in resolution and computational cost, but Grad-CAM remains popular for its balance of simplicity and effectiveness [20].

The present study pursues three objectives. First, we develop a multimodal CNN framework that integrates real-time SDi2 imaging data across two functionally orthogonal wavelengths (280 nm UV channel providing mapping of API release and 520 nm visible channel serving as chemically inert structural probe sensitive to matrix erosion, swelling, and gel-layer formation through refractive index contrast), temporal information, and formulation composition to predict drug dissolution profiles. Second, we employ Grad-CAM to interpret the model’s decision-making process, moving beyond prediction toward a mechanistic understanding. Third, we validate this framework using a set of well-characterized formulations designed to exhibit diverse dissolution behaviors based on API solubility, excipient function, and pH. To this end, compendial buffers (0.1 N HCl, pH 1.2; phosphate buffer, pH 6.8) and a well-characterized set of salicylate derivatives were employed to isolate formulation-driven mechanisms under controlled conditions.

## 2. Materials and Methods

### 2.1. Materials

Three salicylate derivatives were selected for their differing solubility and ionization properties and their UV-absorbing characteristics, enabling direct SDi2 detection: acetylsalicylic acid (ASA), sodium salicylate, and salicylamide. ASA, a weak acid (pKa 3.5), exhibits pH-dependent solubility, as shown in Table 1 [21,22,23].

Sodium salicylate, the ionized salt of salicylic acid, exhibits strongly pH-dependent solubility (0.735 mg/mL at pH 1.2 vs. 812.1 mg/mL at pH 6.8), while salicylamide remains practically un-ionized and sparingly soluble at both test pH values (~2.1 mg/mL). These APIs were formulated with lactose (a soluble filler) or methylcellulose (a gel-forming polymer) to create eight distinct tablet formulations. Dissolution studies were conducted under compendial conditions (0.1 N HCl, pH 1.2; phosphate buffer, pH 6.8) using SDi2 (Pion, Forest Row, UK), generating dual-wavelength imaging data at 280 nm (API absorption) and 520 nm (structural visualization).

### 2.2. Tablet Formulation and Preparation

A total of eight tablet formulations were developed for this study. Each tablet contained 100 mg of the active pharmaceutical ingredient (API) combined with 200 mg of either lactose or methylcellulose (MC) as the primary excipient, along with a 9 mg lubricant blend consisting of talc and magnesium stearate. Additionally, placebo formulations were prepared using only the excipient (200 mg of lactose or MC) and the lubricant blend. All components were manually blended in a mortar, followed by mixing in a plastic container for 5 min to ensure homogeneity. The resulting powder mixtures were compressed into tablets using a single-punch tablet press (Erweka EK0, Langen, Germany) equipped with a 10 mm flat-faced punch. Tablet quality was assessed based on hardness, thickness, and weight uniformity. Tablet hardness was measured using a Pharma Test PTB 311E hardness tester (Pharma Test AG, Hainburg, Germany) and reported in kiloponds (kp); five tablets were analyzed per batch. Thickness measurements were conducted using a digital caliper (Mitutoyo Absolute, Tokyo, Japan) with an accuracy of ±0.01 mm, also on five randomly selected tablets per batch. Uniformity of mass was evaluated by individually weighing each tablet using a precision balance (Mettler Toledo MS105DU, ±0.01 mg, Greifensee, Switzerland).

### 2.3. Dissolution Testing and Real-Time Imaging

Dissolution experiments and real-time imaging were performed using the Pion SDi2™ system (Forest Row, UK), which incorporates a high-resolution CMOS camera (2448 × 2048 pixels), dual-wavelength LED detection (280 nm and 520 nm), and a temperature-controlled flow cell maintained at 37 ± 0.5 °C. The SDi2 operates by directing LED illumination through a quartz flow cell in which the tablet is held stationary; spatially resolved absorbance maps are generated frame-by-frame as dissolution medium flows continuously over the tablet surface, exploiting the Beer-Lambert relationship between local drug concentration and UV absorbance [24,25]. Two compendial media were used: 0.1 N HCl (pH 1.2) and phosphate buffer (pH 6.8), each prepared at a total volume of 900 mL. The dissolution medium was continuously circulated through the SDi2 flow cell at a flow rate of 8 mL/min, regulated by a peristaltic pump (Ismatec IPC-N). Data acquisition consisted of continuous dual-wavelength video capture at 10 s intervals over 240 min testing period, generating raw data of approximately 25 GB per experiment. The UV wavelength of 280 nm was selected from the instrument’s available LED set (255, 280, 300, and 320 nm; bandpass ±5 nm) based on the absorption characteristics of the three model APIs across both test pH values; the visible channel (520 nm) was retained as a structurally sensitive, API-transparent probe.

Sampling protocol:

1. Tablets were secured in a custom stainless-steel holder to prevent movement during testing.
2. Dissolution Initiation: Media (900 mL) was circulated through the flow cell, with continuous magnetic stirring (150 rpm, IKA RCT Basic).
3. Sampling: 5 mL of medium was withdrawn at 0, 5, 10, 15, 30, 45, 60, 120, 180, and 240 min. The removed volume was replaced with fresh medium to maintain sink conditions.

UV spectrometry

Calibration Curves: Six concentrations (2.0–40.0 µg/mL) of each API were prepared in dissolution media. Absorbance was measured at (ASA: 228 nm, Sodium Salicylate: 303 nm, Salicylamide: 299 nm) using a Shimadzu UV-1900 spectrophotometer (Kyoto, Japan). ASA solutions were monitored for hydrolysis (formation of salicylic acid) by tracking absorbance shifts over 4 h in both dissolution media.

### 2.4. Machine Learning Model Development

**Figure 1:**
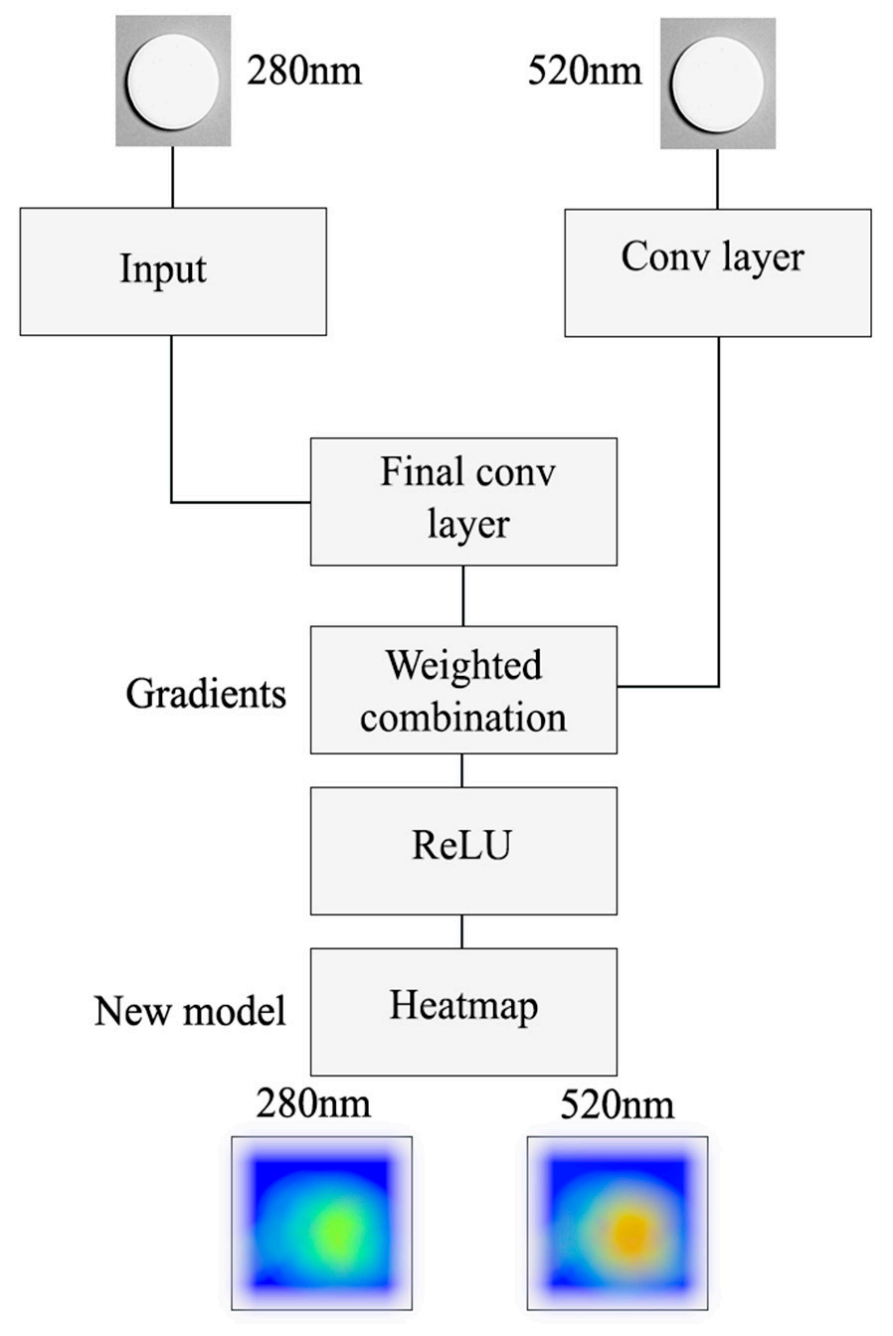
Schematic representation of the custom multimodal CNN architecture. The model integrates dual-wavelength SDi2 images (280 nm and 520 nm), a 224 × 224 × 1 temporal tensor encoding normalized dissolution time, and formulation composition. Inputs are processed through parallel convolutional branches, concatenated in a central fusion layer, and passed through a dense network to predict the dissolved API percentage (Q%). ReLU: rectified linear unit.

The dataset for the model was structured with three distinct input modalities and a single target output. The primary inputs consisted of the dual-wavelength image data. Images captured at 280 nm, corresponding to API release, were processed as 224 × 224 × 3 RGB images. These images were normalized to a [0, 1] range using standard ResNet50 preprocessing procedures. A parallel input stream was established for the 520 nm images, which visualize structural changes; these images were processed identically to maintain the same dimensions and normalization. To provide essential temporal context, a “Time Matrix” was engineered as a third input. To maintain spatial compatibility with the spectral images, this was structured as a 224 × 224 × 1 tensor where every element uniformly encoded the normalized dissolution time point for each image, spanning the full 0–240 min experiment. This input was subsequently processed by a 2D convolutional layer (16 filters, 3 × 3 kernel, ReLU). The model was trained to predict a single continuous target variable per image–time-point input: the dissolved API percentage (Q%), which served as the ‘ground truth’ derived from the offline UV spectrophotometry analysis. A full dissolution profiles are reconstructed by aggregating predictions across all sampled time points. CNN architecture scheme is presented in Figure 1.

This temporal tensor was processed by a dedicated Time Branch, which consisted of a 2D convolutional layer (16 filters, 3 × 3 kernel, ReLU) followed by a 2 × 2 max-pooling layer and a flatten operation. The outputs from this branch, along with the outputs from the parallel image-processing branches, were then fed into a common Fusion Layer^2^. This layer first concatenated the outputs from all branches and then passed the resulting unified vector through a dense network head, which consisted of two hidden layers (128 and 64 neurons, respectively, both using ReLU activation) and a final output layer with a single neuron and a linear activation function to predict the Q% value. For the training protocol, the model was compiled using the Adam optimizer with a learning rate of 0.001, beta_1 of 0.9, and beta_2 of 0.999. The Mean Squared Error (MSE) was selected as the loss function to quantify the model’s prediction error. The complete dataset comprised 153 image–time-point records (8 formulations tested in 2 pH conditions and sampled in 7–10 time points), which were randomly split into an 80:20 train–test ratio (n = 122 for training, n = 31 for testing) using a fixed random seed. Data augmentation techniques were intentionally omitted, as preliminary tests showed that artificial pixel perturbations distort the quantitative absorbance gradients encoded in SDi2 images. To justify the custom architecture, three modelling strategies were evaluated: ImageNet-pretrained ResNet50V2 as a feature extractor, partial fine-tuning of ResNet50V2, and the proposed custom multimodal CNN. All computations were performed using Python 3.10.12. The machine learning pipeline utilized TensorFlow 2.15.0 with Keras 2.15.0 for model development and training, scikit-learn 1.3.2 for data splitting and evaluation metrics, NumPy 1.26.4 for numerical operations, pandas 2.0.3 for data management. A fixed random seed of 1234 was applied throughout all stochastic operations to ensure reproducibility.

### 2.5. Grad-CAM Implementation

To interpret the model’s decision-making process, Grad-CAM (Gradient-weighted Class Activation Mapping) was implemented using TensorFlow’s GradientTape [26]. Gradients of the predicted Q% value were extracted with respect to the final convolutional layer of each image branch (280 nm and 520 nm, respectively), and global average pooling was applied to obtain channel-specific importance weights. The resulting heatmaps were superimposed on the original SDi2 images for visualization (OpenCV v4.8.0, α = 0.5) [27]. Model performance was quantified using R^2^ and RMSE (scikit-learn v1.3.2).

## 3. Results

For each formulation, five tablets were randomly selected from each batch for testing. The thickness and hardness were measured for lactose-based, methylcellulose-based (MC), and placebo formulations. In the lactose-based formulations, the placebo tablets exhibited a mean hardness of 13.92 kp (SD = 0.76), whereas those containing API showed hardness of 6.34 kp (SD = 0.64). A similar trend was noted in the MC-based formulations. The placebo tablets demonstrated a hardness of 20.58 kp (SD = 1.95), compared to 14.38 kp (SD = 0.32) for the API-containing tablets. The thickness measurements for the MC placebo, 3.34 mm (SD = 0.133), and API-MC tablets, 3.20 mm (SD = 0.033). These findings highlight that while the API substantially reduces tablet hardness in both lactose and MC formulations, it does not significantly alter tablet thickness.

### 3.1. Dissolution Profiles

Significant variations in dissolution profiles were observed across formulations, attributable to differences in the dissolution medium pH, excipient composition, and API physicochemical properties. The type of excipient used significantly affected dissolution rates. Lactose promoted rapid surface-initiated release, while methylcellulose sustained drug release through gel-layer formation. The distinct solubility and ionization properties of each API further governed the overall release kinetics in combination with the excipient matrix. The resulting dissolution profiles presented in Table 2 illustrate these differences across the tested formulations.

The equilibrium aqueous solubility of all three APIs in the dissolution media was determined experimentally in our laboratory by UV spectrophotometry using the shake-flask method (20 °C, n = 3). Sodium salicylate exhibited strongly pH-dependent solubility: 0.735 ± 0.003 mg/mL in pH 1.2 buffer (0.1 N HCl), corresponding to the intrinsic solubility of salicylic acid under common-ion conditions (pKa = 2.97; ionized fraction ~1.7%), and 812.1 ± 4.1 mg/mL in phosphate buffer at pH 6.8, where the compound is essentially fully ionized (~99.98%). This 1105-fold increase is consistent with the Henderson–Hasselbalch equation and directly underpins the markedly faster dissolution of sodium salicylate formulations at intestinal pH. Salicylamide (pKa = 8.2) remained practically un-ionized at both test pH values, with measured solubilities of 2.06 ± 0.01 mg/mL at pH 1.2 and 2.14 ± 0.01 mg/mL at pH 6.8, confirming that its dissolution rate is governed by excipient matrix and wettability effects rather than ionization state. Acetylsalicylic acid (ASA; pKa = 3.5) likewise showed pH-independent solubility: 3.89 ± 0.01 mg/mL at pH 1.2 and 4.20 ± 0.02 mg/mL at pH 6.8, indicating that dissolution of ASA-containing tablets is rate-limited by the excipient matrix rather than by drug solubility. All solubility values are summarized in Table 1.

Salicylamide exhibits unique behaviors that significantly impact the role of excipients in tablet formulations. Salicylamide’s solubility and molecular structure can introduce internal stress within the tablet, potentially causing faster API release. This stress originates from molecular-level conformational changes that occur during compaction, particularly when the salicylamide molecule ionizes to form an oxyanion. This ionization leads to a rearrangement of the intramolecular hydrogen bonding network, altering the molecule’s planarity and crystal packing [28]. These molecular strains are then translated into macroscopic residual stress during the tablet compression and decompression process [29,30]. When combined with excipients like methylcellulose (MC), a gelling agent with a viscosity of 400, this effect can be altered. MC forms a gel layer upon contact with dissolution media, creating a barrier that can modulate API release. The gel layer from MC can either facilitate or hinder the penetration of the dissolution medium. In some cases, it enhances tablet erosion and speeds up the dissolution rate. Lactose is a soluble filler whose disintegration behavior in direct compression tablets is primarily wettability-controlled: the rate of liquid penetration through the compact pore network governs the onset of API release [6]. Studies on direct compression formulations confirm that the polymorphic composition of lactose and its pore size distribution are the principal determinants of tablet disintegration time, with lactose monohydrate grades producing faster disintegration than amorphous or anhydrous forms due to their lower initial solubility and reduced propensity for pore-blocking recrystallisation [5]. In our formulations, the rapid, edge-initiated dissolution of lactose-based tablets is consistent with this wettability-controlled mechanism. Another comparison highlights the influence of the API itself, even when the same excipients and pH are used. Table 3 demonstrates this by comparing the release profiles of acetylsalicylic acid and sodium salicylate from identical MC-based matrices.

Under identical MC matrices and acidic medium (0.1 N HCl, pH 1.2), the observed difference between acetylsalicylic acid (ASA; pKa ≈ 3.5) and sodium salicylate can be rationalized by matrix-controlled release rather than bulk solubility alone. At pH 1.2, both APIs are predominantly un-ionized and exhibit comparable aqueous solubility (3.89 vs. 0.735 mg/mL, respectively); the faster release of ASA therefore reflects differences in molecular interactions with the MC matrix and the erosion front dynamics rather than a solubility-driven effect.

Despite having identical excipients, different APIs can result in significant variations in dissolution behavior, emphasizing the critical role that the API plays in the formulation process. In contrast to comparing APIs, Table 4 illustrates the critical impact of the excipient by comparing the release of salicylamide from a gelling (MC) versus a soluble (lactose) matrix.

Interestingly, salicylamide released faster from MC-based than from lactose-based tablets at pH 1.2, suggesting that factors beyond simple excipient solubility govern the release kinetics in this system. The underlying mechanisms warrant further investigation.

### 3.2. SDi2 Dissolution Analysis

**Figure 2:**
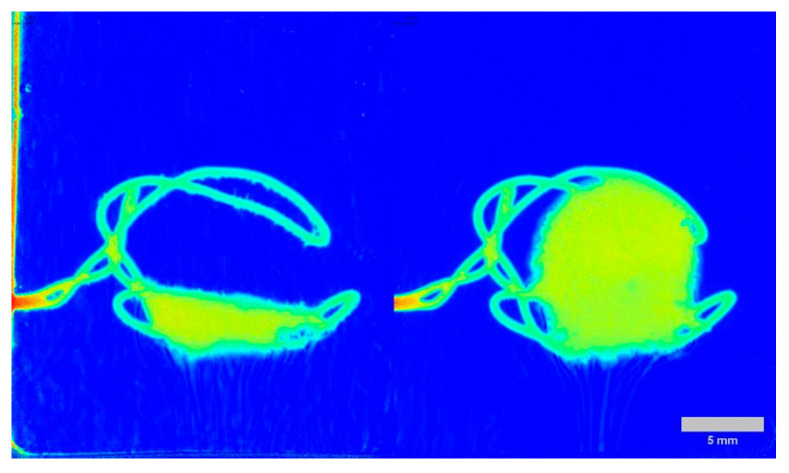
SDi2 images (520 nm). Left: placebo lactose tablet at pH 1.2 after 5 min; Right: sodium salicylate/lactose tablet at pH 1.2 at the same time point. Medium: 0.1 N HCl, 900 mL, 37 ± 0.5 °C. The stainless-steel tablet holder is visible as the mounting bracket. The grey scale bar represents 5 mm. The spatial distribution of absorbance is visualized using a jet colormap, where dark blue indicates zero absorbance (background), and the progression towards warmer colors (green and yellow) represents increasing absorbance values.

Eight formulations were tested, with each formulation analyzed at two different wavelengths (280 nm and 520 nm) under varying pH conditions (1.2 and 6.8). Dissolution profiles were captured in real-time, with 5 mL of each sample taken at the following time intervals: 5, 10, 15, 30, 45, 60, 120, 180, and 240 min. The complete time-lapse image sets gathered at 520 nm, which visually document these distinct structural changes for lactose-based formulations under both acidic (pH 1.2) and neutral (pH 6.8) conditions, are presented in the Supplementary Materials (Tables S1 and S2). Placebo formulations were also included in the study to assess the impact of the API on the tablet behavior. One possible explanation for the results is that the API affects the entire disintegration and dissolution process, potentially due to differences in solubility or chemical interactions occurring in the mixture (Figure 2).

**Figure 3:**
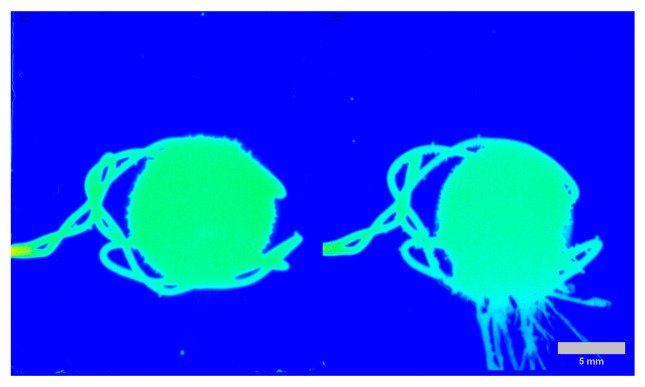
SDi2 images (520 nm) at 30 min, pH 1.2. Left: sodium salicylate with lactose (rapid erosion/IR); Right: sodium salicylate with MC showing diffusion gel layer and slower release. Medium: 0.1 N HCl; 900 mL; 37 ± 0.5 °C; Flow: 8 mL/min. The stainless-steel tablet holder is visible as the mounting bracket. The grey scale bar represents 5 mm. The spatial distribution of absorbance is visualized using a jet colormap, where dark blue indicates zero absorbance (background), and the progression towards warmer colors (green and yellow) represents increasing absorbance values.

Excipients significantly influence the dissolution behavior between different formulations, particularly when comparing lactose and methylcellulose (MC). Lactose-based tablets exhibit immediate-release (IR) behavior, dissolving rapidly at pH 1.2, while MC provides extended release by forming an erosion gel layer. This gel layer helps protect the tablet in the harsh acidic environment until it reaches pH 6.8, where it releases the API. This theory was supported by the experimental results. In the case of lactose tablets, the API was released after 30 min, whereas in the MC-based tablet, the API remained encapsulated due to the protective gel layer. Figure 3 below demonstrates this process.

### 3.3. Predictive Models Performance

To evaluate the predictive power of our approach, we developed and tested a series of models with increasing complexity. Our goal was to determine the contribution of each data modality—imaging, time, and composition—to the final prediction accuracy. As confirmed by the code, all models were validated using a consistent 80:20 train–test split, and all reported performance metrics (R^2^ and RMSE) were calculated on the 20% held-out test set, confirming the models’ ability to generalize to unseen data. This process began with a baseline model, a Dual-Wavelength CNN, which took only the images (280 nm and 520 nm) as input. This architecture consisted of two parallel convolutional branches (3 Conv2D layers each), which were fused via concatenation before being passed to the final dense layers. This model achieved moderate performance on the test set, yielding an R^2^ of 0.75 and an RMSE of 17.09, with the best result achieved at 50 epochs.

Next, to contextualize the visual data, this model was expanded to include temporal data, which was encoded as a 2D “time matrix.” This three-input network, also utilizing a concatenation layer to fuse the image and time branches, showed a significant improvement, achieving an R^2^ of 0.83 and an RMSE of 14.01 on the test set. The final, most comprehensive model integrated all available data modalities: 280 nm images, 520 nm images, the time matrix, and a one-hot encoded formulation composition vector. This architecture combines all four input branches via concatenation, processing them through common dense layers. This complete model achieved the best performance, yielding a test set R^2^ of 0.89 and an RMSE of 11.57 (best result at 200 epochs). For a full comparison, the DNN model was also trained using numerical data (time and composition). This simpler model still achieved results (R^2^ = 0.86, RMSE = 12.96), highlighting the predictive value of the formulation data alone.

### 3.4. Predicted vs. Observed Values

**Figure 4:**
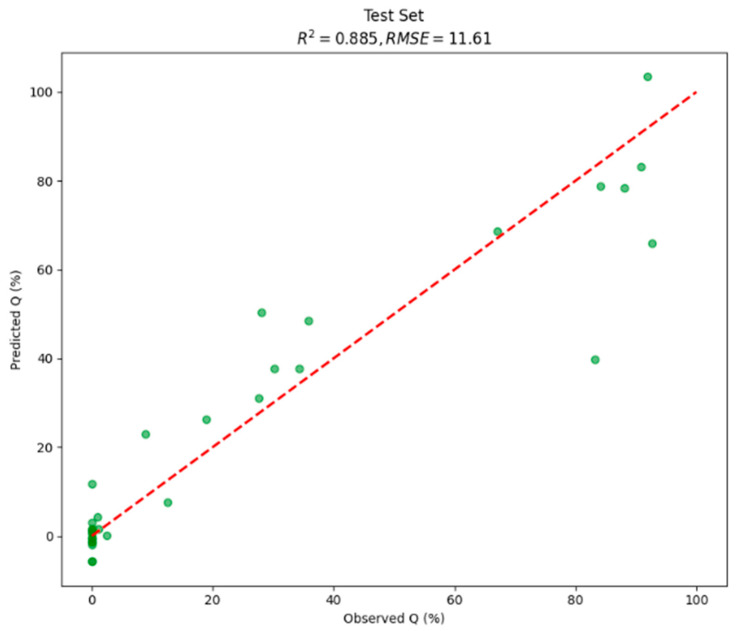
Scatter plot presenting predicted vs. observed values of multimodal CNN on test set (20% of total data). The dashed line denotes the identity (perfect prediction).

**Figure 5:**
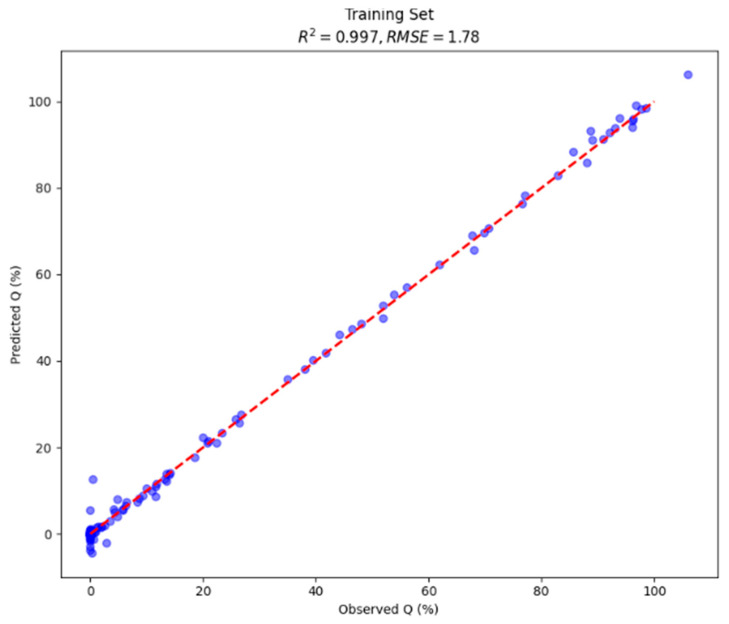
Scatter plot presenting predicted vs. observed values of multimodal CNN on train set (80% of total data). The dashed line denotes the identity (perfect prediction).

**Figure 6:**
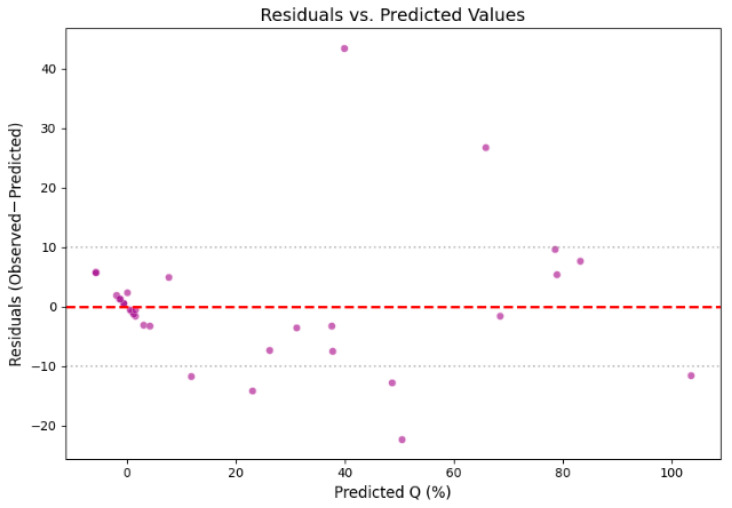
Residual plot for the held-out test set: difference between predicted and measured Q% (predicted−observed) as a function of measured Q%. The dashed line at zero represents perfect prediction. Residuals are distributed randomly around zero with no systematic trend.

To evaluate the predictive accuracy of the final multimodal CNN, model-predicted Q% values were compared against reference UV-Vis measurements on both the training set and the held-out test set. Figure 4, Figure 5 and Figure 6 present scatter plots of predicted versus observed values and the residuals.

Moreover, stratified analysis performed within the existing held-out test set (n = 31; pH 1.2: n = 16, pH 6.8: n = 15) revealed R^2^ = 0.957 and RMSE = 7.21% at pH 1.2 and R^2^ = 0.804 and RMSE = 14.94% at pH 6.8. For reference, a linear regression model trained on an identical data split using dissolution time and one-hot encoded formulation composition as inputs achieved R^2^ = 0.278 and RMSE = 29.14% on the test set.

### 3.5. Grad-CAM Insights

**Figure 7:**
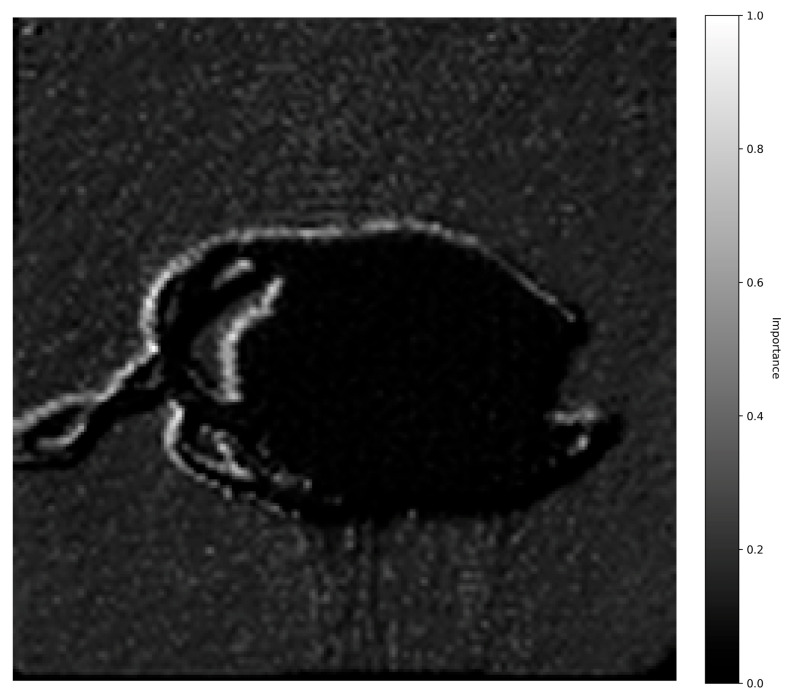
280 nm Grad-CAM heatmaps. Activation maps highlighting regions most influential for predicting API release. High-importance (importance = gradient-weighted pixel contribution to predicted Q%) zones appear at the tablet edges and the tablet–medium interface, corresponding to early dissolution and surface erosion sites.

The 280 nm Grad-CAM heatmap (Figure 7) shows intense activation at the tablet edges and the tablet–medium interface, indicating that the model focused on the primary API release zones. In ASA/lactose tablets, this activation was asymmetric, confirming that the model identified the leading tablet edge most exposed to media flow as the dominant dissolution front, a finding consistent with the wettability-controlled, edge-initiated erosion mechanism characteristic of lactose-based formulations.

**Figure 8:**
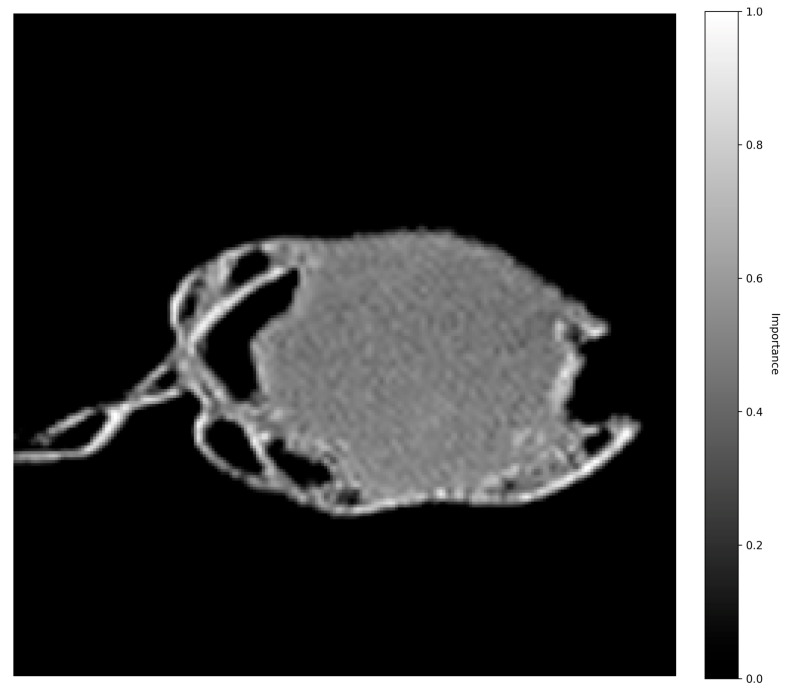
520 nm Grad-CAM heatmaps. Activation maps showing model attention on structural changes, such as pore formation and gel-layer development. The CNN focuses primarily on the tablet core, reflecting deeper matrix transformations during dissolution. The heatmap intensity denotes the gradient-weighted contribution of each pixel to the predicted Q%, highlighting regions most influential for the model’s prediction.

The 520 nm heatmap (Figure 8) is focused on the tablet core, revealing structural changes like pore formation and gel layer expansion. In both wavelength analyses, significant activation was also noted on the stainless-steel tablet holder.

It is important to distinguish these Grad-CAM results from standard SDi2 imaging. While Figure 2 and Figure 3 display raw visual data of the physical dissolution process, the heatmaps in Figure 7 and Figure 8 represent gradient-based saliency maps of the CNN, where the ‘importance’ axis quantifies the gradient-weighted contribution of each pixel to the model’s prediction of dissolved API percentage (Q%). Notably, activation was also observed on the stainless-steel tablet holder visible in the images. While the mechanistic basis of this activation requires further investigation, it may reflect the model using the static bracket as a spatial reference against which tablet boundary changes are implicitly quantified. Overall, the Grad-CAM activation patterns are presented as model-transparency findings confirming that the CNN attends to physically plausible image regions. The 520 nm core activations are qualitatively consistent with the established role of the visible channel in detecting matrix structural changes through refractive index contrast [24,31].

## 4. Discussion

The integration of advanced imaging with artificial intelligence represents a new frontier in pharmaceutical sciences, and this study introduces a novel framework that combines surface dissolution imaging (SDi2) with a dual-wavelength convolutional neural network (CNN) to both predict and interpret the complex dissolution behavior of oral solid dosage forms. The central achievement is the development of a highly accurate predictive model, which synergistically integrates dual-wavelength images with temporal and formulation data to achieve a coefficient of determination (R^2^) of 0.89 and a root mean square error (RMSE) of 11.57. This compares favorably with CNN-based approaches applied to static chemical imaging, such as the fast Raman mapping method of Galata et al. [13], which achieved R^2^ = 0.87 on held-out test tablets, and with the real-time UV imaging CNN framework of Stróżyk et al. [17], which reported R^2^ = 0.84 for in vitro–in vivo relationship modelling. The improvement over the latter baseline is attributable to the addition of the second imaging wavelength (520 nm) and the explicit formulation-composition input branch, both of which provided information orthogonal to time-resolved dissolution kinetics. The model’s success further stems from its capacity for multimodal data fusion, where the incremental addition of temporal and compositional data clearly enhanced predictive power.

While predictive accuracy is critical, a primary contribution of this work is overcoming the “black-box” nature of deep learning through the application of Gradient-weighted Class Activation Mapping (Grad-CAM), an explainable AI (XAI) technique. Practically, these activation maps function as a spatial design guide. Edge-focused importance at 280 nm, observed for lactose-based formulations, identifies surface wettability and disintegrant performance as the rate-controlling design variables, suggesting that modifications to particle size, surface area, or surfactant content would be the most effective optimization targets. Conversely, core-focused importance at 520 nm, characteristic of MC matrices, identifies gel-layer thickness and viscosity as the dominant rate-limiting factors, pointing to polymer concentration and grade as the key formulation levers. Grad-CAM thus enables a rational, evidence-based transition from empirical formulation screening toward Quality-by-Design (QbD) optimization. The Grad-CAM analysis revealed that the model independently learned to associate each imaging wavelength with its correct physical phenomenon. When processing images at 280 nm, attention was concentrated on the tablet edges and the tablet-fluid interface, the zones of API release. Conversely, at 520 nm, its focus shifted to the tablet’s core and bulk structure, where macroscopic changes occur. The model’s differential focus correctly identified the rapid, surface-initiated disintegration of lactose-based tablets and the slow, bulk-controlled release from methylcellulose matrices, which is governed by the formation of a rate-limiting gel layer [5,32].

Situating this work in the broader landscape of pharmaceutical AI, our methodology represents a methodological advance. While significant research has successfully used static, pre-dissolution chemical maps from techniques like Raman imaging to predict release, our approach utilizes in situ, real-time imaging, which fundamentally changes the nature of the predictive task. Instead of surrogate modeling, the model learns the temporal evolution of the system, effectively creating a data-driven simulation of the physical process. The feasibility of this dynamic approach was established in foundational work by Stróżyk et al. [17], presenting the use of real-time UV imaging and CNNs for modeling in vitro-in vivo relationships. The present research builds directly upon that foundation and significantly enhances the paradigm by introducing a dual-wavelength system. This critical enhancement allows the model to deconvolve two distinct but concurrent processes for the first time: the chemical event of API release, tracked at 280 nm, and the physical transformation of the excipient matrix, visualized at 520 nm. This multi-faceted, dynamic analysis provides a more granular and mechanistically rich understanding than was previously possible, marking a significant step forward in the field. However, the study’s limitations must be acknowledged, including the need to validate the model on a wider chemical space. Future work should focus on expanding the training dataset to encompass a wider chemical and formulation space, which would further strengthen the generalizability of the proposed framework. Additionally, the spatially resolved nature of Grad-CAM activations presents an interesting opportunity for future experimental follow-up, linking model attention patterns to measurable changes within the tablet matrix. The present study extends beyond existing imaging-based dissolution modelling approaches in several respects. While Galata et al. [16] demonstrated CNN-based prediction from static pre-dissolution Raman maps of a single-formulation matrix, and Stróżyk et al. [17] showed the feasibility of SDi2-based IVIVR modelling, neither approach combined real-time dual-wavelength dissolution imaging with quantitative Q% prediction across a heterogeneous (various APIs) multi-formulation space. Similarly, complementary approaches using combined UV/Raman spectroscopy [33] or ANN-based prediction from bulk spectra [34] do not offer a unified predictive–interpretive framework operable directly from raw dissolution images, further contextualising the methodological differences from the present contribution.

The regulatory landscape for AI in pharmaceutical development is evolving rapidly. The FDA issued draft guidance on AI in regulatory decision-making in January 2025, and the FDA and EMA jointly published guiding principles for good AI practice in drug development in January 2026, emphasizing risk-based validation, transparency, and context-of-use definitions. The present framework is intended for exploratory use in early-stage formulation development rather than quality control or batch release; under the risk-based approach promoted by both agencies, such applications carry a substantially lower regulatory burden than deployment in regulated QC settings.

## 5. Conclusions

This study presents a framework combining SDi2 with a multimodal CNN to predict and interpret dissolution behavior of oral solid dosage forms. Our work achieved its threefold objective: first, to develop a multimodal CNN framework for predicting API release from SDi2 imaging data; second, to apply Grad-CAM to decode the CNN’s decision-making process, thereby correlating its predictions with tangible physicochemical mechanisms like gel layer formation and surface erosion; and third, to leverage these integrated insights for a more informed approach to formulation optimization. The resulting final model, which integrated dual-wavelength images with temporal and formulation data, achieved competitive performance (R^2^ = 0.89, RMSE = 11.57), demonstrating generalization to unseen data. Crucially, the use of Grad-CAM highlighted which regions of the tablet surface or core dominated the release behavior (e.g., edge erosion for lactose vs. gel-layer thickening for MC), suggesting that explainable AI can help formulation scientists identify which structural features most influence dissolution and therefore guide excipient selection and matrix design. Key findings confirmed that the model learned to recognize how excipients like lactose and methylcellulose alter release kinetics, with MC formulations exhibiting sustained release due to gel layer formation. The framework also effectively captured how APIs with pH-dependent solubility, such as ASA, produce variable release profiles in different compendial media. This level of discernment was made possible by the dual-wavelength approach, which simultaneously captures both surface API release and bulk structural phenomena, enabling far more accurate predictions than could be achieved with single-wavelength methods. By integrating real-time imaging with explainable AI, this study provides a proof-of-concept framework for data-driven dissolution analysis, suggesting potential utility in formulation screening and early-stage development. However, broader applicability will require validation on larger and more chemically diverse datasets, encompassing a wider range of APIs, excipients, and dosage form designs, before generalizing the findings to real-world pharmaceutical development contexts.

## Figures and Tables

**Table 1:** Physicochemical properties and ionization constants of the model APIs.

Compound	pKa	Ionization at pH 1.2	Solubility at pH 1.2 [mg/mL]	Solubility at pH 6.8 [mg/mL]
Acetylsalicylic acid	3.5	Predominantly un-ionised	3.89 ± 0.01 **	4.20 ± 0.02 **
Sodium salicylate	3.0 *	Predominantly un-ionised	0.735 ± 0.003 **	812.1 ± 4.1 **
Salicylamide	8.2	Un-ionised	2.06 ± 0.01 **	2.14 ± 0.01 **

* pKa of parent salicylic acid; ** own experimental data; APIs: active pharmaceutical ingredients.

**Table 2:** Dissolution profiles of sodium salicylate formulations in pH 1.2 medium, comparing lactose-based and methylcellulose (MC)-based matrices.

Time [min]	API Released (Lactose-Based) [%]	API Released (MC-Based) * [%]
5	2.8	0.5
10	12.5	2.6
15	20.8	4.1
30	44.1	8.8
45	61.8	11.7
60	69.8	14.1
120	N/A **	23.2
180	N/A **	30.2
240	N/A **	35.8

* MC: Methyl cellulose, ** N/A: Sampling was terminated as the lactose-based tablet had completely disintegrated before this time point. Note: Values represent a single SDi2 dissolution experiment.

**Table 3:** Dissolution profiles of acetylsalicylic acid and sodium salicylate from MC-based tablets, both at pH 1.2.

Time	% ASA * Released	% NaSalicylate *** Released
5	0.2	0.5
10	4.2	2.6
15	11.6	4.1
30	35.0	8.8
45	67.0	11.7
60	82.8	14.1
120	90.9	23.2
180	91.0	30.2
240	91.9	35.8

* ASA: acetylsalicylic acid, *** NaSalicylate: sodium salicylate, MC: methyl cellulose. Values represent a single SDi2 dissolution experiment.

**Table 4:** Dissolution profiles of salicylamide tablets with lactose or MC at pH 1.2.

Time [min]	MC-Based Tablet * [%]	Lactose-Based Tablet [%]
5	0.0	0.1
10	1.0	1.1
15	4.8	2.4
30	25.7	6.2
45	56.1	10.0
60	76.6	13.4
120	88.1	26.7
180	88.0	39.5
240	88.6	46.4

* MC: Methyl cellulose. Values represent a single SDI2 dissolution experiment.

## Data Availability

The dataset is available on request.

## Supplementary Materials

The following supporting information can be downloaded at: https://www.mdpi.com/article/10.3390/pharmaceutics18040481/s1. Table S1: SDi2 time-lapse images (520 nm) for lactose-based formulations in pH 1.2 medium; Table S2: SDi2 time-lapse images (520 nm) for lactose-based formulations in pH 6.8 medium.

## Abbreviations

The following abbreviations are used in this manuscript:

API	Active Pharmaceutical Ingredient
ARA	Acid-Reducing Agent
ASA	Acetylsalicylic Acid
AUC	Area Under the Curve
BCS	Biopharmaceutics Classification System
CAM	Class Activation Mapping
Cmax	Maximum Concentration of Drug in Plasma
CNN	Convolutional Neural Network
DDI	Drug–Drug Interaction
DNN	Deep Neural Network
GI	Gastrointestinal
Grad-CAM	Gradient-weighted Class Activation Mapping
HCl	Hydrochloric Acid
HPMC	Hydroxypropyl Methylcellulose
KCl	Potassium Chloride
KH_2_PO_4_	Potassium Dihydrogen Phosphate
LRP	Layer-wise Relevance Propagation
MC	Methylcellulose
MSE	Mean Squared Error
NaSa	Sodium Salicylate
NaOH	Sodium Hydroxide
OpenCV	Open Source Computer Vision Library
PEO	Polyethylene Oxide
PPI	Proton Pump Inhibitor
Q%	Percentage of API dissolved
QbD	Quality-by-Design
ReLU	Rectified Linear Unit
RGB	Red Green Blue
RKinetDS	Software for Modeling Dissolution Profiles
RMSE	Root Mean Square Error
SaAmide	Salicylamide
SDi2	Surface Dissolution Imaging
TensorFlow	Machine Learning Framework
USP	United States Pharmacopeia
UV–Vis	Ultraviolet–Visible Spectroscopy
