# Supplementary material for: Explainable AI in Pharmaceutics: Grad-CAM Analysis of Surface Dissolution Imaging Using Convolutional Neural Networks
Source: Pharmaceutics. 2026 Apr 14;18(4):481. doi: 10.3390/pharmaceutics18040481 (PMC13119162; doi:10.3390/pharmaceutics18040481)
Supplement: Supplementary file 1 [file pharmaceutics-18-00481-s001.zip › Table S1.pdf]

Table S1: Absorbance of Tablet Formulations (with lactose) at 520 nm in pH 1.2 Medium Over Time

| Time | Placebo (Lactose) | Lactose(NASA) | LactoseASA | Lactose(Salicylamide) |
|---|---|---|---|---|
| 0min | 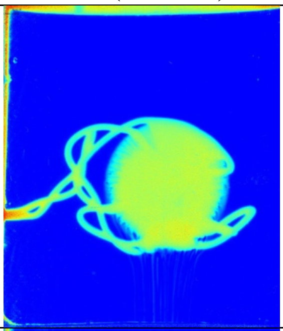 | 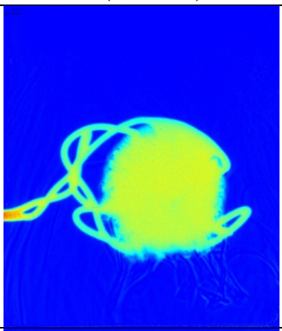 | 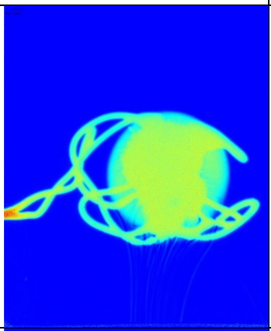 | 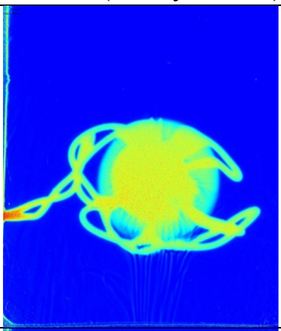 |
| 5min | 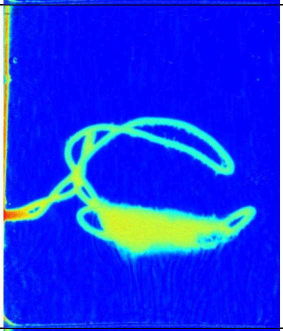 | 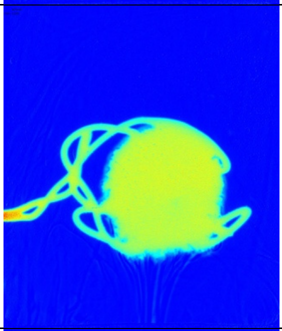 | 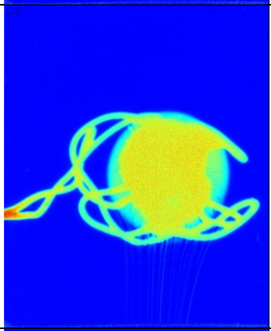 | 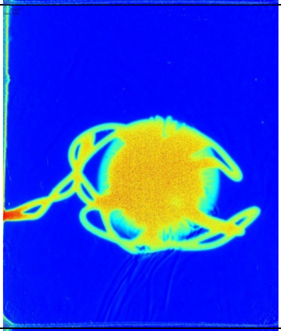 |
| 10min | 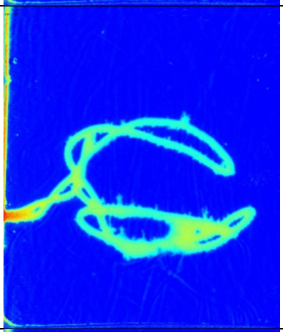 | 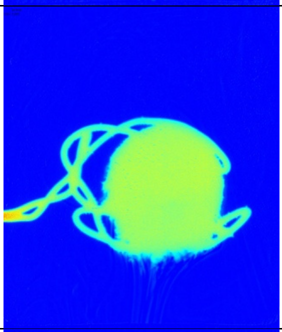 | 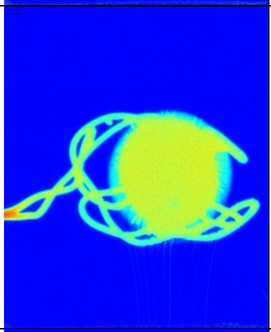 | 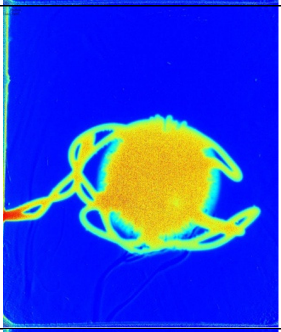 |
| 15min | 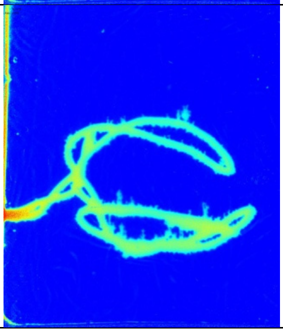 | 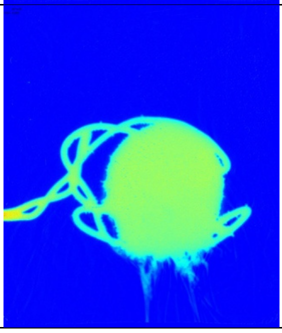 | 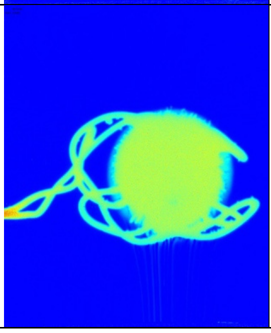 | 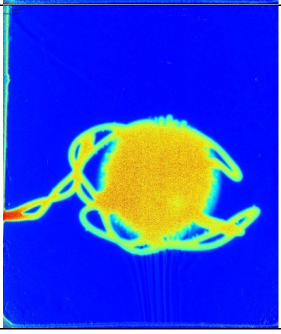 |

| | | | | |
|---|---|---|---|---|
| 30min | 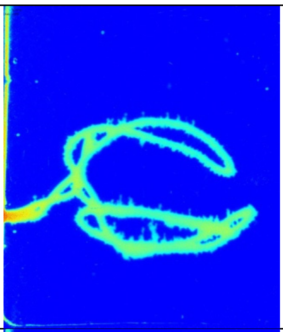 | 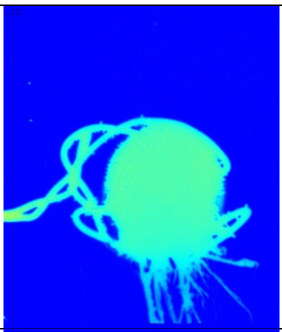 | 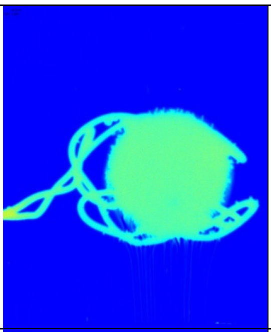 | 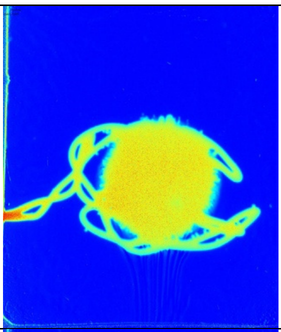 |
| 45min | 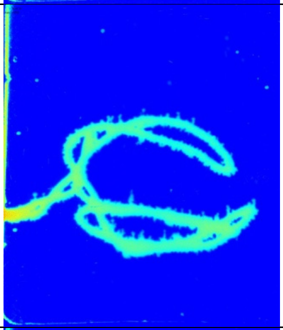 | 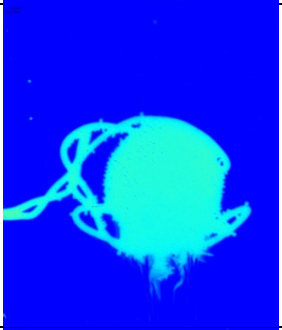 | 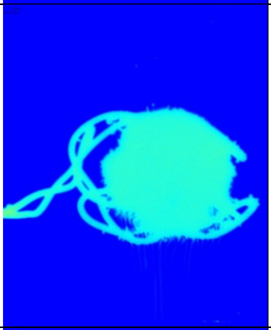 | 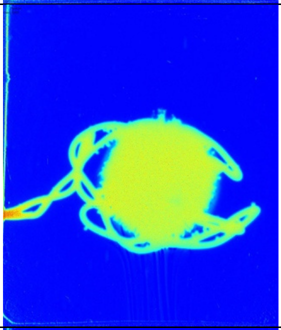 |
| 60min | 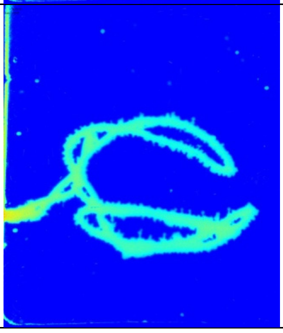 | 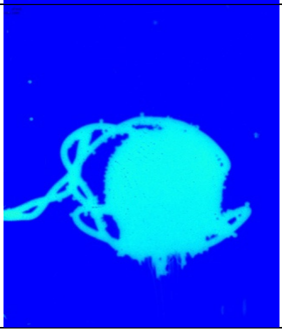 | 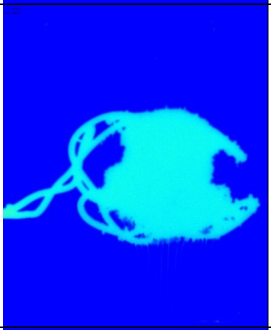 | 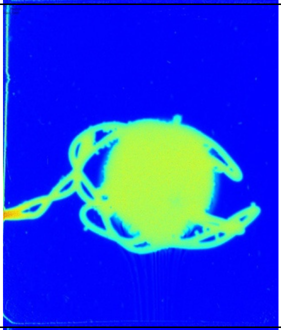 |
| 120min | ND | ND | 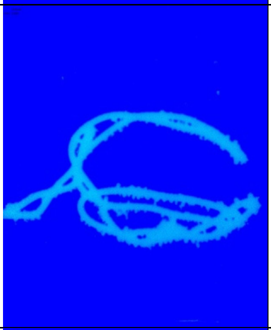 | 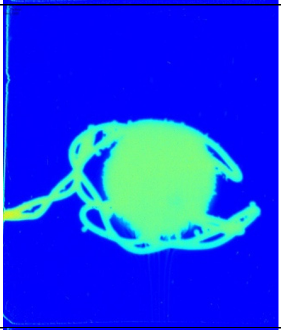 |
| 180min | ND | ND | 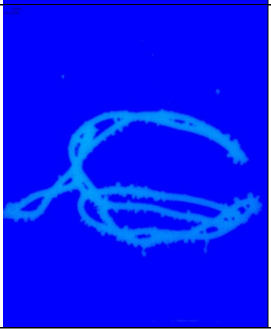 | 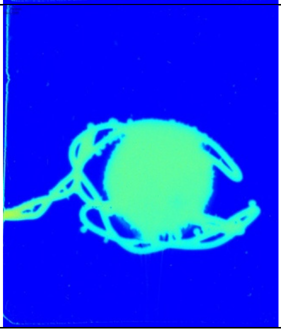 |

| | | | |
|---|---|---|---|
| 240min | ND | ND | 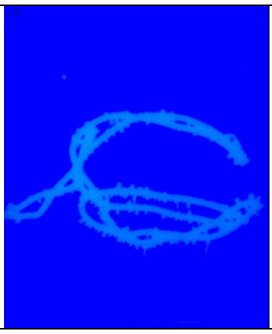 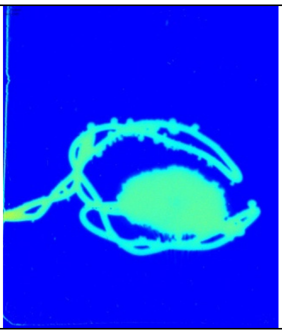 |

*ND: Not Detected
