# Supplementary material for: Explainable AI in Pharmaceutics: Grad-CAM Analysis of Surface Dissolution Imaging Using Convolutional Neural Networks
Source: Pharmaceutics. 2026 Apr 14;18(4):481. doi: 10.3390/pharmaceutics18040481 (PMC13119162; doi:10.3390/pharmaceutics18040481)
Supplement: Supplementary file 1 [file pharmaceutics-18-00481-s001.zip › Table S2.pdf]

Table S2: Absorbance of Tablet Formulations (with lactose) at 520 nm in pH 6.8 Medium Over Time

Time	Placebo (Lactose)	Lactose + NaSA	Lactose + ASA	Lactose + SA
0min	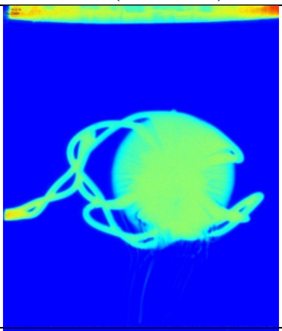	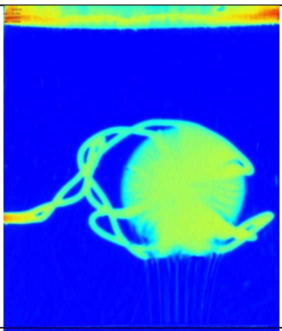	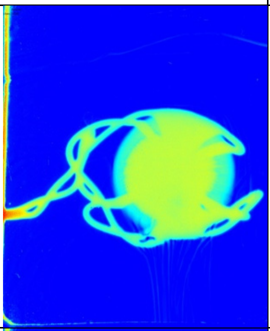	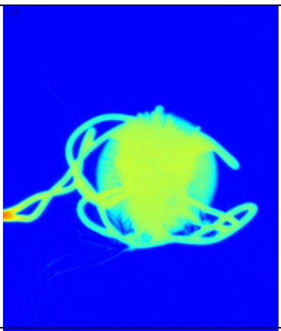
5min	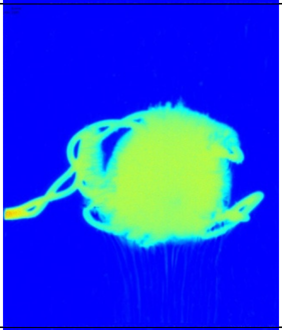	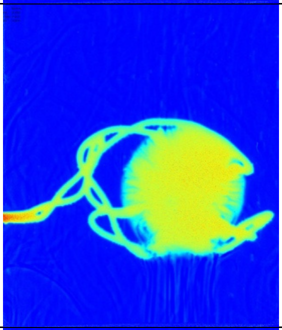	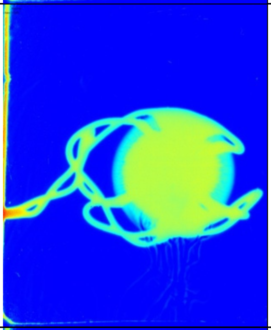	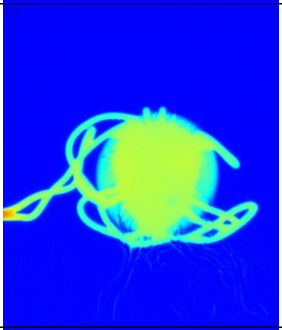
10min**	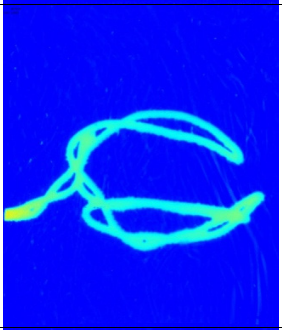	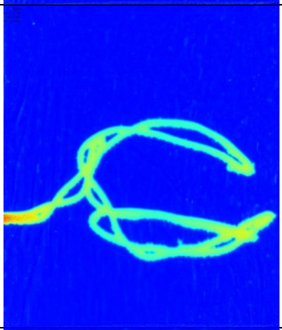	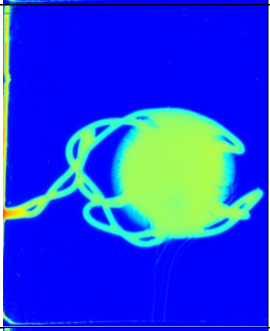	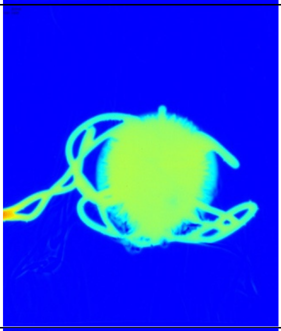
15min	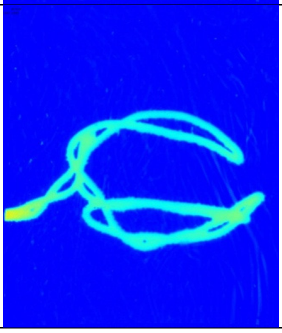	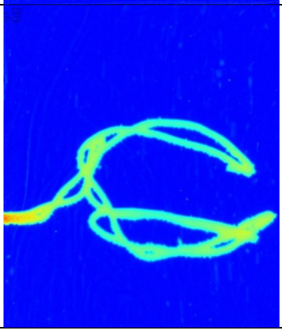	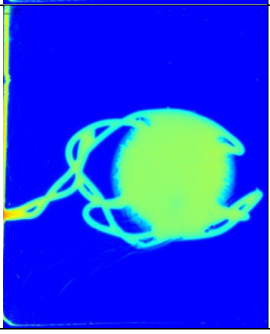	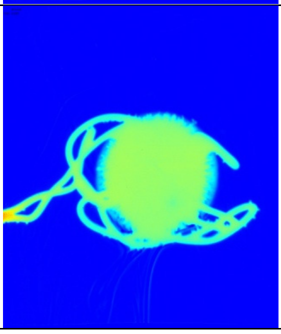

30min	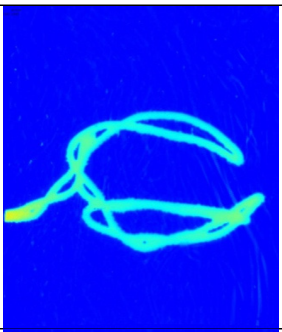	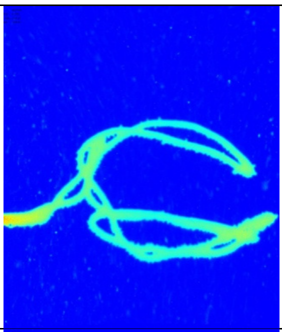	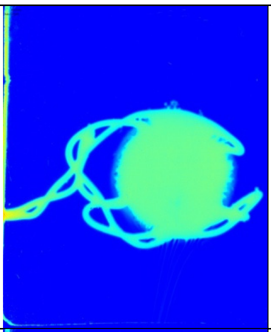	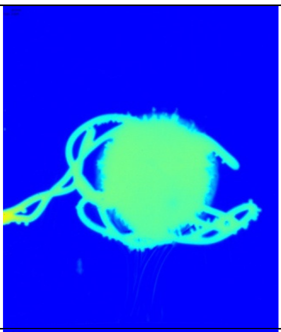
45min	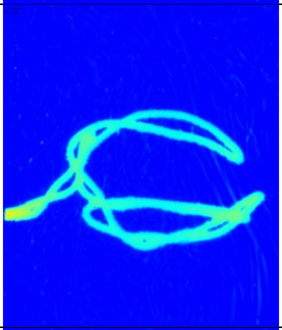	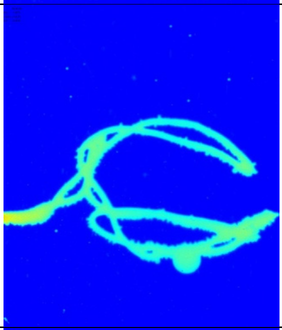	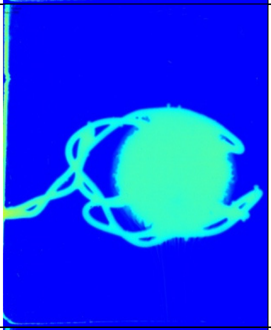	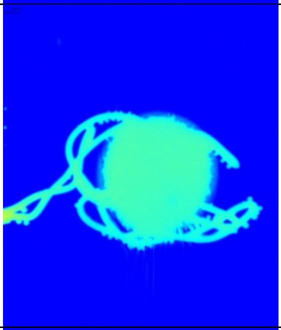
60min	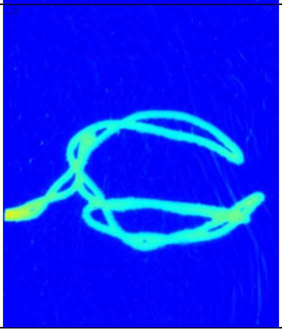	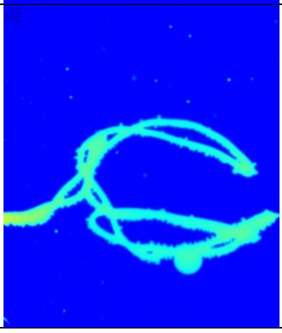	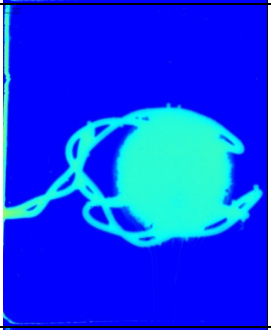	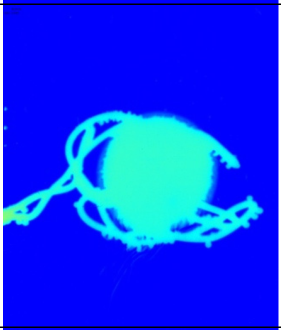
120min			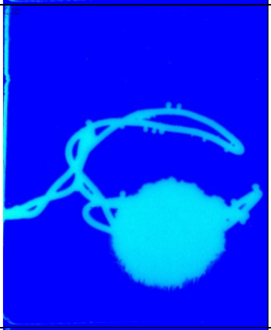	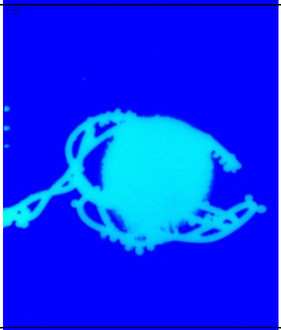
180min			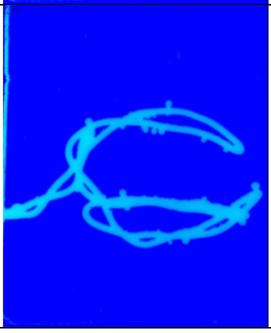	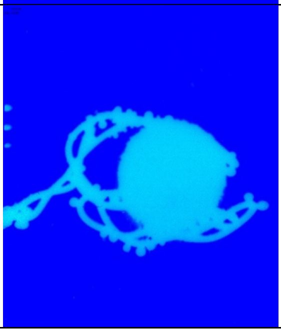

240min			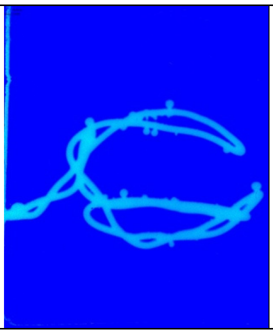 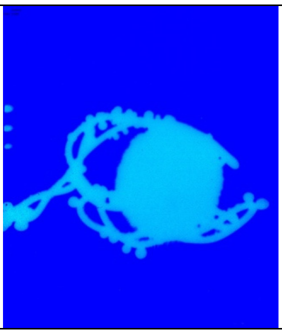
